# Leveraging Novel Methods to Chart New Horizons in the Study of Personality, Stress, and Cognitive Aging

**DOI:** 10.1093/geroni/igaf122.276

**Published:** 2025-12-31

**Authors:** Eileen Graham, Emorie Beck

## Abstract

In order to optimize health in older adulthood, it is crucial to understand how variability in modifiable psychosocial factors fluctuate both short- and long-term. Using several innovative methodological approaches, this symposium highlights how psychosocial processes (e.g., stress, personality) that unfold across multiple time scales are critical for cognitive aging. First, Beck examines individual differences in cognitive coupling (i.e. momentary, within-person associations among cognitive domains) in later life and associations with sociodemographic and risk factors. Second, Ma explores associations between momentary Big Five personality states and cognitive function levels and variability using mixed effects location scale models, with preliminary results suggesting that personality states impact cognitive variability as much as momentary cognitive function. Third, Zavala explores whether believing stress can serve as an enhancer (i.e. stress mindset) and perceived stress are associated with momentary cognitive function. Preliminary findings indicate that older adults tended to report less momentary perceived stress but that neither stress mindset nor momentary perceived stress were associated with cognitive performance. Finally, Luo will present a coordinated analysis that explores how Big Five personality traits are associated with longitudinal patterns of social stress (i.e., social asymmetry) in multiple longitudinal studies of aging. Preliminary findings suggest that individuals who are higher in neuroticism become more socially vulnerable over the lifespan, while those higher in extraversion, conscientiousness, and agreeableness become more socially resilient. We will close with a panel discussion about the utility of investigating psychosocial and cognitive processes across multiple timescales for promoting health aging.

